# A case series of atypical presentation of Zika Virus infection in Singapore

**DOI:** 10.1186/s12879-016-2032-y

**Published:** 2016-11-17

**Authors:** Bang Yu Xu, Sher Guan Low, Richard Tiong Heng Tan, Farhad Fakhrudin Vasanwala

**Affiliations:** Department of Family Medicine, Sengkang Health, SingHealth, Alexandra Hospital, 378 Alexandra Road, Singapore, Singapore 159964

**Keywords:** Case series, Case definition, Atypical presentation, Zika, Outbreak, Singapore

## Abstract

**Background:**

The World Health Organization’s and Centers for Disease Control and Prevention’s definition of Zika infection are symptoms of fever, rash, joint pain, myalgia, headache and conjunctivitis. The diagnosis of Zika infection is based on the clinical history, physical examination and laboratory investigations which includes blood and urine Zika virus Polymerase Chain Reaction.

**Case presentation:**

Two patients presented with atypical presentation of Zika infection to Sengkang Health, Alexandra Hospital during the recent Zika outbreak in Singapore in August 2016.

Madam A presented with isolated generalized rash with no fever, joint pain, myalgia, headache or conjunctivitis.

Mr. B presented with isolated fever of 39.4 °C with no rash, joint pain, myalgia, headache or conjunctivitis.

Both patients’ blood Zika Polymerase Chain Reactions were positive at the time of presentation.

**Conclusion:**

The described case reports illustrated the challenges that our community Family Physicians faced in diagnosing patients infected with Zika virus. Coupled with the knowledge that most patients are asymptomatic, Family Physicians need to have a high index of clinical suspicion for early identification of patients infected with Zika virus, so as to institute timely treatment and appropriate measures to mitigate the outbreak of Zika infection in the community. Appropriate epidemiological measures such as ensuring prompt and thorough contact tracing of the cases are instrumental in the control of this public health problem.

## Background

With the recent surge in cases of Zika infection in South America and other countries like Cambodia and Thailand, WHO has declared that Zika virus is currently a global public health emergency. As Singapore is a major transport hub, it is inevitable that cases of Zika virus are imported to Singapore. There is also a high risk of local transmission due to the endemic presence of Aedes mosquitoes in Singapore.

In Singapore, MOH has been closely monitoring the Zika virus situation and has introduced several measures to improve the surveillance of the disease and the protection of Singaporeans. Singaporeans are also advised to take precautionary measures to prevent mosquitoes breeding, thus emphasizing heavily on vector control as a critical component. To prevent Zika virus from becoming entrenched in our local population, MOH also undertook strict control measures. All confirmed symptomatic cases, as indicated by positive blood Zika PCR, were admitted to a public hospital until they recovered and were tested negative for the virus. The authorities in MOH also carried out active surveillance, looking for additional cases in high risk geographical regions with areas in relation to the confirmed cases. The first case of locally transmitted Zika virus infection was reported on 27th August 2016.

Most patients with Zika virus infection were either asymptomatic or had mild symptoms. According to CDC, the most common symptoms of Zika infection are fever, rash, joint pain, conjunctivitis [1]. Other encountered symptoms may include myalgia and headache. The duration of symptoms may range from a few days to a week. Based on CDC recommendation, diagnosis of Zika is based on a person’s recent travel history, symptoms, and test results [2]. The serum or urinary Zika PCR can confirm Zika infection.

In Singapore, MOH case definition of Zika virus infection is as follows [3] :Individuals presenting with fever **AND** maculopapular rash **AND** any of the following:

- Arthralgia (Joint Pain)
- Myalgia (Muscle Pain)
- Headache
- Non-Purulent Conjunctivitis

- Point to note:

Any individual with a recent travel history (within 2 weeks) to Zika-affected areas [4]

We propose that the criteria above serve only as a guide and further field studies need to be done to define the case definition of this disease.

## Cases presentation

### Case 1

Madam A was a 53 years old lady, with previous history of total hysterectomy with bilateral salpingo-oophorectomy performed in 2013 for uterine fibroids. She presented with only generalized rash for one day. She did not complain of fever, joint pain, myalgia, headache or conjunctivitis. She also did not have other localizing symptoms suggestive of infection. She noticed a mosquito bite about three days prior to the onset of the rash. She stayed at the Eastern region of Singapore, which was identified as a Zika-affected area in Singapore. She did not have any recent overseas travel history.

She came across information about Zika infection from an educational poster and hence sought medical consultation at Tan Tock Seng Hospital Accident and Emergency Department. On examination, she was well and clinically not dehydrated. Her temperature was 36.8 °C, blood pressure was 139/76 mmHg, pulse rate was regular at 82/min, and respiratory rate of 18/min with an oxygen saturation of 98% on room air. A generalized maculopapular rash was seen. Examination of her cardiorespiratory systems were unremarkable. Examination of her abdomen was unremarkable as well, with no evidence of hepatosplenomegaly. There were no lymphadenopathies detected during the physical examination.

The initial laboratory investigations revealed a normal white blood cell counts with normal neutrophils and lymphocytes counts, slightly raised monocytes count at 1.14 [normal range: 0.20 - 0.70 (x10^9/^L)]; normal haemoglobin and platelet counts. Dengue NS1 antigen, Dengue IgM and IgG levels were not detected. Her Urea, Creatinine and Electrolytes test and Liver Function Test were normal. Blood and Urine Zika PCR were positive. All these tests were done one day after the appearance of the generalized rash. Diagnosis of Zika virus infection was confirmed and she was admitted to Sengkang Health @ Alexandra Hospital for further management.

During her in-patient stay, she remained relatively well. She did not develop any fever, myalgia, arthralgia, conjunctivitis or headache. Her physical examination was unremarkable, with only the presence of generalised maculopapular rash. On second day of hospitalisation, her repeat Full Blood Count was normal, with no drop in her platelet counts. Her repeat blood Zika PCR was negative and urine Zika PCR was still positive. As she was clinically well and her serum Zika PCR was negative, Madam A was deemed fit to be discharged from the hospital. During her stay, she was educated about the Zika virus and its mode of transmission and vector control measures. Advice was also given to seek medical consult should she develop any additional symptoms.

A phone consult was made two days later to check on Madam A. She had completely recovered, with resolution of her generalised maculopapular rash and she remained well.

### Case 2

Mr. B was a 30 years old gentleman, with background history of fatty liver and obesity on follow-up with his Family Physician. He presented with the solitary symptom of fever for two days. His highest recorded temperature was 39.4 °C. He did not have any localising symptoms to suggest any source of infection. He also did not complain of any myalgia, arthralgia, headache, conjunctivitis or rashes. He did not come into contact with anyone with similar symptoms and did not have any recent overseas travel. He stayed at Central region of Singapore [4], which was identified as a Zika-affected area in Singapore and his Family Physician was very concerned about the possibility of Zika infection due to the rising incidence of cases in the geographical vicinity. Thus, he referred Mr. B to Tan Tock Seng Hospital (TTSH) Accident and Emergency Department for Zika PCR testing despite being advised by the TTSH A&E staff that it was not necessary as it did not fulfill the criteria for referral at that time.

On examination at Tan Tock Seng Hospital Accident and Emergency Department, he was well and clinically not dehydrated. His temperature was 37.7 °C, blood pressure was 131/67 mmHg, pulse rate was regular at 92/min, respiratory rate at 18/min with saturation at 97% on room air. There were no obvious rash seen and the examination of his cardiorespiratory systems were unremarkable. Examination of his abdomen was unremarkable as well, with no evidence of hepatosplenomegaly. There were no lymphadenopathies detected during the physical examination.

The initial laboratory investigations revealed normal white blood cell counts with normal neutrophils, slight reduced lymphocytes counts at 0.75 [normal range: 0.90 - 3.30 (x10^9^/L)], slightly raised monocytes count at 1.02 [normal range: 0.20 - 0.70 (x10^9^/L)]; normal haemoglobin and platelet counts. Dengue NS1 antigen, Dengue IgM and IgG levels are not detected. His Urea, Creatinine and Electrolytes test and Liver Function Test were normal. Blood and Urine Zika PCR were positive. All these tests were done on the second day of his fever presentation. Diagnosis of Zika virus infection was confirmed and he was admitted to Sengkang Health @ Alexandra Hospital for further management.

During his in-patient stay, he was given symptomatic treatment for his fever. He did not develop any rashes, myalgia, arthralgia, conjunctivitis or headache during the first two days of his hospitalization stay. He remained afebrile throughout his hospitalization stay. On the third day of his stay, he developed flushing of both his upper limbs which progressed to a generalised flushing affecting his trunk, limbs and face. His physical examination was otherwise unremarkable, with only the presence of generalised maculopapular rash. Daily Full Blood Counts were performed and his platelet counts remained stable. Daily serum Zika PCR were performed to trend for progression. On the fourth day of his stay, his serum Zika PCR was noted to be negative while urine Zika PCR remained positive. With a negative serum Zika PCR and clinical improvement, Mr. B was deemed fit to be discharged from the hospital. During his stay, he was educated about the Zika virus and its mode of transmission and vector control measures. Advice was also given to seek medical consult should he develop any additional symptoms.

## Conclusion

The described case reports illustrated the challenges that our community Family Physicians faced in diagnosing patients infected with Zika virus. Coupled with the knowledge that most patients are asymptomatic, Family Physicians need to have a high index of clinical suspicion for early identification of patients infected with Zika virus, institute treatment and measures to reduce the outbreak of Zika infection in the community. Family Physicians play an important role at the Primary Healthcare level in educating the public that vector control remains key to reducing the spread of the Zika virus. Residents should undertake vector control measures regularly to ensure proper housekeeping within their premises at all times and remove any potential mosquito breeding habitats. Residents also need to do their part to prevent mosquito breeding in their homes by doing the five-step Mozzie Wipeout [5].

We believe that there may be more cases of patients that did not present typically and thus did not fit into the WHO and CDC case definitions, and thus might have been missed. Thus, more field reports of the presentation of the disease will be necessary to develop a more robust definition of this disease, especially within the South East Asia Region. The possibility that the Asian strain of the Zika virus may present differently from that of the South American strain needs to be further explored. There are public health implications especially for those couples planning to start families. It will be important to be aware of such atypical presentations of this disease as the child conceived by such unsuspecting mothers may develop serious developmental delays. Judicious allocations of resources can thus be made to uncover such cases who might be pregnant and presented atypically, for screening of birth developmental defects during their pregnancies.

## Abbreviations

CDC: Centers for Disease Control and Prevention
Dengue NS1 antigen: Dengue non-structural protein 1 antigen
EMR: Electronic medical record
MOH: Ministry of Health
PCR: Polymerase chain reaction
WHO: World Health Organization
